# Ti_3_C_2_T*_x_*/MoS_2_ Self‐Rolling Rod‐Based Foam Boosts Interfacial Polarization for Electromagnetic Wave Absorption

**DOI:** 10.1002/advs.202201118

**Published:** 2022-04-11

**Authors:** Minghang Li, Wenjie Zhu, Xin Li, Hailong Xu, Xiaomeng Fan, Hongjing Wu, Fang Ye, Jimei Xue, Xiaoqiang Li, Laifei Cheng, Litong Zhang

**Affiliations:** ^1^ Science and Technology on Thermostructural Composite Materials Laboratory Northwestern Polytechnical University Xi'an 710072 P. R. China; ^2^ Institute of Textiles and Clothing The Hong Kong Polytechnic University Hong Kong SAR 999077 P. R. China; ^3^ MOE Key Laboratory of Material Physics and Chemistry under Extraordinary School of Physical Science and Technology Northwestern Polytechnical University Xi'an 710072 P. R. China

**Keywords:** electromagnetic wave absorption, heterogeneous interfaces, MoS_2_, MXene foams, self‐rolling rod

## Abstract

Heterogeneous interface design to boost interfacial polarization has become a feasible way to realize high electromagnetic wave absorbing (EMA) performance of dielectric materials. However, interfacial polarization in simple structures such as particles, rods, and flakes is weak and usually plays a secondary role. In order to enhance the interfacial polarization and simultaneously reduce the electronic conductivity to avoid reflection of electromagnetic wave, a more rational geometric structure for dielectric materials is desired. Herein, a Ti_3_C_2_T*_x_*/MoS_2_ self‐rolling rod‐based foam is proposed to realize excellent interfacial polarization and achieve high EMA performance at ultralow density. Different surface tensions of Ti_3_C_2_T*_x_* and ammonium tetrathiomolybdate are utilized to induce the self‐rolling of Ti_3_C_2_T*_x_* sheets. The rods with a high aspect ratio not only remarkably improve the polarization loss but also are beneficial to the construction of Ti_3_C_2_T*_x_*/MoS_2_ foam, leading to enhanced EMA capability. As a result, the effective absorption bandwidth of Ti_3_C_2_T*_x_*/MoS_2_ foam covers the whole *X* band (8.2–12.4 GHz) with a density of only 0.009 g cm^−3^, at a thickness of 3.3 mm. The advantages of rod structures are verified through simulations in the CST microwave studio. This work inspires the rational geometric design of micro/nanostructures for new‐generation EMA materials.

## Graphical abstract

A more rational geometric structure for dielectric materials, i.e., a self‐rolling rod‐based foam structure is proposed, which can not only remarkably enhance the interfacial polarization loss but also simultaneously reduce the electronic conductivity to avoid reflection of electromagnetic wave. This work will inspire the rational geometric design of micro/nanostructures for new‐generation electromagnetic wave absorbing materials.

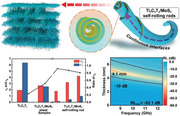

## 1 Introduction

Spatial electromagnetic (EM) wave pollution has been an unavoidable issue in daily life with the rapid development of electronic technology. To reduce EM wave interference, extensive studies about EM wave absorbing (EMA) materials have been propelled.^[^ ^1^ ^]^ Generally, the rational design of heterogenous interface in dielectric EMA materials can optimize the impedance match conditions (Table S1, Supporting Information).^[^ ^2^ ^]^ As a result, the optimal heterogeneous interface design has become a mainstream way to realize high EMA performance.^[^ ^3^ ^]^

2D material Ti_3_C_2_T*_x_* MXene is often used to carry on interface design due to its high specific surface areas and solution‐processibility.^[^ ^4^ ^]^ However, the pristine Ti_3_C_2_T*_x_* possesses high electrical conductivity (higher than 10 000 S cm^−1^), leading to impedance mismatch and showing EM interference (EMI) shielding performance.^[^ ^5^ ^]^ For instance, Ti_3_C_2_T*_x_* foam with a density of 5.5 mg cm^−3^ shows shielding effectiveness of 54.8 dB.^[^ ^6^ ^]^ To optimize the impedance match condition, heterogeneous interface design based on Ti_3_C_2_T*_x_* has been carried out, such as Ti_3_C_2_T*_x_*/SiC, Ti_3_C_2_T*_x_*/NiCo_2_O_4,_ and so on.^[^ ^7^ ^]^ Although the EMA performances have been improved to some extent in these structures, the interfacial polarizations are still weak in these traditional sheet structures. Due to the limit of sheet structures, it is hard to increase the number of interfaces between two sheets. Recent research verifies that the rod structure is one of the most effective structures to enhance polarization loss, such as core–shell or core–sheath.^[^ ^8^ ^]^ Nevertheless, in these structures, the interface structure between core and shell is very simple. Self‐rolling rod structures without cores are expected to overcome the above problems and realize the great increase of interfaces between two materials. The interfaces between two materials will increase greatly with the decrease of the diameter of this self‐rolling rod structure. Furthermore, the electronic conductivity of Ti_3_C_2_T*_x_* will decrease rapidly due to the composition with the second phase such as semiconductor in the self‐rolling rod structure, which could optimize the impedance match conditions. However, there are still synthetic methodological challenges to the implementation of self‐rolling rod structures for Ti_3_C_2_T*_x_*.

Except for microstructure, macroscopic foam structures are usually adopted as one of the most promising structures for EMA materials, whose high porosity facilitates the entry and transmission of EM wave inside samples and is beneficial to the dissipation of EM wave energy.^[^ ^1^, ^8^, ^9^ ^]^ Self‐rolling rod structures with a high aspect ratio are expected to efficiently construct EMA foams, due to the formation of intertwined networks during the process of the freeze‐drying method.^[^ ^10^ ^]^ Therefore, self‐rolling rod structures are not only helpful for constructing foam structures but also good for the formation of massive heterogeneous interfaces between two phases. Based on the above analysis, it is an efficient way to realize lightweight and high EMA capability by constructing MXene‐based foam with self‐rolling rod structures.

**Figure 1:**
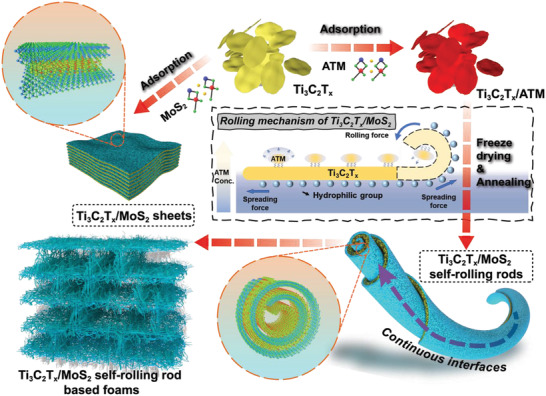
Schematic illustration of the fabrication process of Ti_3_C_2_T*_x_*/MoS_2_ sheets and self‐rolling rods and rolling mechanism of Ti_3_C_2_T*_x_*/MoS_2_ self‐rolling rods.

In this work, the 3D foams composed of Ti_3_C_2_T*_x_*/MoS_2_ self‐rolling rods were prepared through the unidirectional freeze‐drying method (**Figure** **1**). Ammonium tetrathiomolybdate (ATM) was used as the precursor of MoS_2_. The different surface tension directions of ATM and Ti_3_C_2_T*_x_* would induce the self‐rolling of Ti_3_C_2_T*_x_*/MoS_2_.^[^ ^11^ ^]^ There is a big jump in the number of interfaces after the self‐rolling process and the interfaces between Ti_3_C_2_T*_x_* and MoS_2_ are utilized to increase the interfacial polarization. As a result, the ratio of polarization loss remarkably reaches above 80% and the impedance match condition is greatly improved. The effective absorption bandwidth (EAB) covers the whole *X* band and can cover *C* and *Ku* bands according to simulation results. To investigate the influence of the ratio of Ti_3_C_2_T*_x_*/MoS_2_, three Ti_3_C_2_T*_x_*/MoS_2_ self‐rolling rod samples named S1–S3 were prepared by adjusting the contents of Ti_3_C_2_T*_x_* and MoS_2_. The EMA mechanisms of Ti_3_C_2_T*_x_*/MoS_2_ self‐rolling rod structures are studied through simulation in the CST microwave studio.

## 2 Results and Discussion

### 2.1 Formation Mechanism and Micro/Nanostructure

Figure 1 illustrates the fabrication process and rolling mechanism of Ti_3_C_2_T*_x_*/MoS_2_ foam. Ti_3_C_2_T*_x_* sheets were prepared through in situ fluohydric acid (HF) etching and hand‐shaking method.^[^ ^4c^ ^]^ The abundant surface groups (—F, —O, and —OH) on the Ti_3_C_2_T*_x_* sheets make them good dispersion properties in an aqueous solution.^[^ ^12^ ^]^ Thus, the surface tension of Ti_3_C_2_T*_x_* sheets tends to flatten themselves (spreading force). However, the dispersion property of the ATM in the aqueous solution is poor. The surface tension of ATM tends to curl the droplets from the edge to achieve minimum surface energy (rolling force).^[^ ^11b^ ^]^ As a result, after attachment with Ti_3_C_2_T*_x_* through Van der Waals' force, the rolling force derived from the surface tension of ATM would induce the self‐rolling of Ti_3_C_2_T*_x_* sheets (Figure 1 and Figure S1, Supporting Information). Figure S2 in the Supporting Information shows that a change in pH value does not result in morphological changes of Ti_3_C_2_T*_x_* sheets, further proving that the surface tension is critical to the rolling of Ti_3_C_2_T*_x_*. In addition, a temperature gradient existed along the vertical direction, benefiting from the fine control of the freezing process. While the aqueous suspension was frozen, the ice crystals grew along the vertical direction and expelled Ti_3_C_2_T*_x_*/ATM rod to the gaps between ice crystals. The orderly lamellar foam structure was constructed after the ice sublimation under vacuum. After annealing at 350 °C for 2 h, the MoS_2_ was formed by the decomposition of the ATM. The thermogravimetry (TG) curve and decomposition equations of ATM are given in Figure S3 in the Supporting Information, which indicates that about 25% of weight loss during annealing, caused by the escape of NH_3_.^[^ ^13^ ^]^ To demonstrate the advantages of self‐rolling rod structure, the Ti_3_C_2_T*_x_*/MoS_2_ sheets were also prepared. The ATM was annealed at first to obtain MoS_2_ and then mixed with Ti_3_C_2_T*_x_* followed by the same unidirectional freezing process.

**Figure 2:**
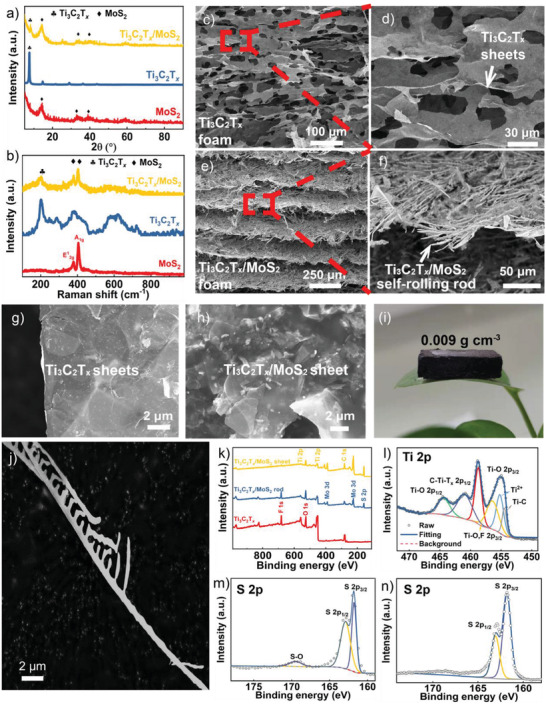
a) XRD and b) Raman patterns of MoS_2_, T_3_C_2_T*_x_*, and Ti_3_C_2_T*_x_*/MoS_2_. Cross‐sectional scanning electron microscope (SEM) images of c,d) Ti_3_C_2_T*_x_* foam and e,f) Ti_3_C_2_T*_x_*/MoS_2_ foam. SEM image of g) Ti_3_C_2_T*_x_* sheets and h) Ti_3_C_2_T*_x_*/MoS_2_ sheets. i) The optical photos of Ti_3_C_2_T*_x_*/MoS_2_ self‐rolling rod‐based foam. j) SEM image of single Ti_3_C_2_T*_x_*/MoS_2_ self‐rolling rod. k) X‐ray photoelectron spectroscopy (XPS) spectra of Ti_3_C_2_T*_x_*, Ti_3_C_2_T*_x_*/MoS_2_ self‐rolling rod structure, and Ti_3_C_2_T*_x_*/MoS_2_ sheet structure. l) High‐resolution of Ti 2p spectrum of Ti_3_C_2_T*_x_*/MoS_2_ self‐rolling rod structure. m,n) High‐resolution of S 2p spectrum of Ti_3_C_2_T*_x_*/MoS_2_ self‐rolling rod structure and Ti_3_C_2_T*_x_*/MoS_2_ sheet structure, respectively.

**Figure 3:**
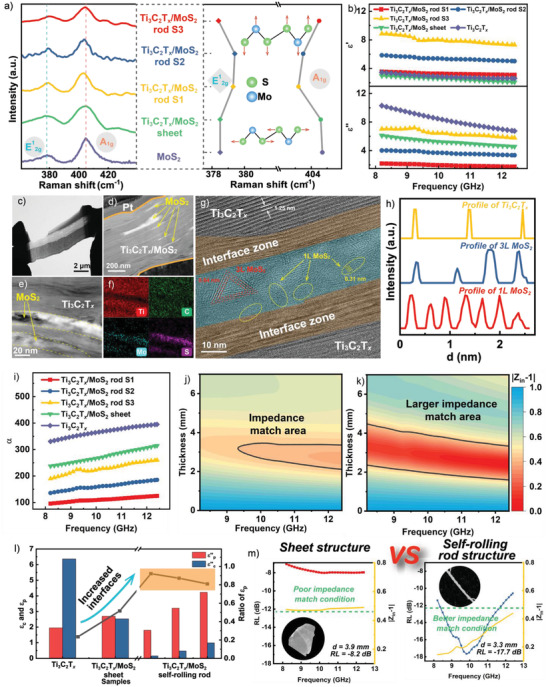
a) E^1^_2g_ mode and A_1g_ mode of Raman spectra of MoS_2_, Ti_3_C_2_T*_x_*/MoS_2_ sheet, and Ti_3_C_2_T*_x_*/MoS_2_ self‐rolling rod. b) The real and imaginary part of permittivities of all samples. c) The cross‐sectional sample of Ti_3_C_2_T*_x_*/MoS_2_ self‐rolling rod prepared by FIB technique and d) its corresponding transmission electron microscope (TEM) images. e,f) The high‐magnification TEM image and its corresponding energy disperse spectroscopy (EDS) mapping images. g) The interface between Ti_3_C_2_T*_x_* and MoS_2_. h) The intensity profiles of 1L MoS_2_, 3L MoS_2,_ and Ti_3_C_2_T*_x_*. i) Attenuation constants of Ti_3_C_2_T*_x_*, Ti_3_C_2_T*_x_*/MoS_2_ sheets, and Ti_3_C_2_T*_x_*/MoS_2_ self‐rolling rods. *|Z*_in_ *−* 1*|* contours of j) Ti_3_C_2_T*_x_*/MoS_2_ sheets and k) Ti_3_C_2_T*_x_*/MoS_2_ self‐rolling rods. l) The conductive and polarization loss of Ti_3_C_2_T*_x_*, Ti_3_C_2_T*_x_*/MoS_2_ sheets, and Ti_3_C_2_T*_x_*/MoS_2_ self‐rolling rods. m) Comparation of EMA performances for Ti_3_C_2_T*_x_*/MoS_2_ sheets and Ti_3_C_2_T*_x_*/MoS_2_ self‐rolling rods, revealing the superiority of self‐rolling rod structure for enhancing EM wave absorption and impedance match condition.

**Figure** **2**a shows the X‐ray diffraction (XRD) patterns of MoS_2_, Ti_3_C_2_T*_x_*, and Ti_3_C_2_T*_x_*/MoS_2_. For the MoS_2_, the peak around 15° represents the (002) crystal plane of MoS_2_, which verifies the decomposition of ATM.^[^ ^14^ ^]^ The peak at 7.4° is the characteristic peak of Ti_3_C_2_T*_x_*, representing the (002) plane of Ti_3_C_2_T*_x_*.^[^ ^15^ ^]^ After heat‐treatment of the Ti_3_C_2_T*_x_*‐ATM sample, the characteristic diffraction peaks of MoS_2_ appear. It is noted that the peak of the (002) plane of Ti_3_C_2_T*_x_* shifts from 7.4° to 6.8°, which means that the d‐spacing of the (002) plane increased from 11.70 to 12.97 Å, which is consistent with the interlayer spacing of Ti_3_C_2_T*_x_* in **Figure** **3**g. It can be concluded that the MoS_2_ sheets were in situ formed in the interlayer of Ti_3_C_2_T*_x_* flakes, leading to the enlarged interlayer space. Raman spectra of MoS_2_, Ti_3_C_2_T*_x_*, and Ti_3_C_2_T*_x_*/MoS_2_ are shown in Figure 2b. Consistent with previous studies, three broad peaks of Ti_3_C_2_T*_x_* are shown at ≈202, 378, and 605 cm^−1^.^[^ ^16^ ^]^ The broad peak at 605 cm^−1^ represents the existence of —OH groups.^[^ ^17^ ^]^ After binding with MoS_2_, the peak of —OH groups disappeared, which means that the ATM attaches with Ti_3_C_2_T*_x_* through —OH groups.

The SEM images in Figure 2c–f show that both Ti_3_C_2_T*_x_* and Ti_3_C_2_T*_x_*/MoS_2_ foams are similar layered structures. The layer spacing of Ti_3_C_2_T*_x_* foam is only 30 µm and the layer spacing of Ti_3_C_2_T*_x_*/MoS_2_ foam is 200 µm. The larger layer spacing is propitious to the entry of EM waves into Ti_3_C_2_T*_x_*/MoS_2_ foam. According to the high magnification SEM images, the layer arrangements of Ti_3_C_2_T*_x_*/MoS_2_ and Ti_3_C_2_T*_x_* are different. The layer of Ti_3_C_2_T*_x_* foam is composed of 2D Ti_3_C_2_T*_x_* sheets and there are some holes on the surface (Figure 2d). The layer of Ti_3_C_2_T*_x_*/MoS_2_ foam is composed of crossed rods (Figure 2f). Compared with Ti_3_C_2_T*_x_* and Ti_3_C_2_T*_x_*/MoS_2_ sheets, the Ti_3_C_2_T*_x_*/MoS_2_ self‐rolling rods cross each other and show a rough surface (Figure 2g,h). The N_2_ sorption isotherms at 77 K and pore‐size distribution derived from Barrett–Joyner–Halenda method are shown in Figure S4 in the Supporting Information. The specific surface areas of Ti_3_C_2_T*_x_*/MoS_2_ sheets and Ti_3_C_2_T*_x_*/MoS_2_ self‐rolling rods are 5.2327 and 3.7463 m^2^ g^−1^, respectively. The self‐rolling structure leads to the decrease of specific surface areas, due to a lot of surfaces being used to construct heterogeneous interfaces. The pore size distributions of Ti_3_C_2_T*_x_*/MoS_2_ sheets and Ti_3_C_2_T*_x_*/MoS_2_ self‐rolling rods are similar. However, the pore volume of Ti_3_C_2_T*_x_*/MoS_2_ self‐rolling rods is 0.016 cm^3^ g^−1^, which is higher than 0.013 cm^3^ g^−1^ of Ti_3_C_2_T*_x_*/MoS_2_ sheets. A larger pore volume is beneficial to the impedance match condition. To better observe the morphology of Ti_3_C_2_T*_x_*/MoS_2_ self‐rolling rods, a single Ti_3_C_2_T*_x_*/MoS_2_ self‐rolling rod was obtained through sonication (Figure 2j). The length of a single rod can reach tens of micrometers and there are some synapses‐like structures along the rod, which are beneficial to the construction of Ti_3_C_2_T*_x_*/MoS_2_ foams. These flexible rods are interconnected through synapses (Figure S5a, Supporting Information), which are like bridges, leading to the formation of stable foam structures (Figure 2i and Figure S9, Supporting Information). The foam structures composed of Ti_3_C_2_T*_x_*/MoS_2_ self‐rolling rods facilitate the entry and transmission of EM wave inside samples and are propitious to the consumption of EM wave energy through heat dissipation.^[^ ^18^ ^]^

The XPS survey in Figure 2k demonstrates four main elements, including Ti, C, O, and F in Ti_3_C_2_T*_x_*. After attaching with MoS_2_, the signal of S and Mo can be detected. The XPS spectrum of Ti 2p in pure Ti_3_C_2_T*_x_* foam shows four predominant peaks of Ti—O 2p_1/2_ (464.3 eV), Ti—O 2p_3/2_ (458.8 eV), Ti—C 2p_3/2_ (455.1 eV), and C—Ti—T*_x_* 2p_1/2_ (461.1 eV) bonds. These chemical states are caused by the chemical etching process of Ti_3_AlC_2_ using HCl and LiF, which can introduce many functional groups.^[^ ^19^ ^]^ The preparation of MoS_2_ is confirmed by the Mo 3d and S 2s peaks. In the S 2p spectrum, the two conspicuous peaks of S 2p_1/2_ and S 2p_3/2_ at 162.9 and 161.8 eV represent the S^2−^ of MoS_2_.^[^ ^20^ ^]^ It is worth noting that there is a small peak at about 169.5 eV for Ti_3_C_2_T*_x_*/MoS_2_ self‐rolling rod structure, which can be assigned to S—O bonds, indicating the combination of MoS_2_ and —O groups of Ti_3_C_2_T*_x_*.^[^ ^21^ ^]^ The S 2p spectrum of Ti_3_C_2_T*_x_*/MoS_2_ shows that there is no S—O bond, which means MoS_2_ and Ti_3_C_2_T*_x_* are just mixed simply. As a result, the signal of O 1s is stronger in Ti_3_C_2_T*_x_*/MoS_2_ compared with pure Ti_3_C_2_T*_x_* due to the partly oxidized MoS_2_ layer on the surface of Ti_3_C_2_T*_x_*.

The Raman spectra of MoS_2_ show two dominant peaks around 400 cm^−1^, which are assigned to E^1^_2g_ and A_1g_ mode of S—Mo—S.^[^ ^22^ ^]^ The frequency difference of E^1^_2g_ and A_1g_ peaks can be used to evaluate the property of MoS_2_ and the distance of Mo—S bonds (Figure 3a). The frequency differences between E^1^_2g_ and A_1g_ peaks are about 26 cm^−1^ for both MoS_2_ and Ti_3_C_2_T*_x_*/MoS_2_ sheets, which are consistent with bulk MoS_2_.^[^ ^23^ ^]^ For Ti_3_C_2_T*_x_*/MoS_2_ self‐rolling rods, the frequency differences between E^1^_2g_ and A_1g_ peaks decrease to about 24 cm^−1^, which are consistent with few‐layer MoS_2_. Apart from that, the E^1^_2g_ mode exhibits a slight blueshift from Ti_3_C_2_T*_x_*/MoS_2_ sheet to Ti_3_C_2_T*_x_*/MoS_2_ self‐rolling rod, suggesting a decrease of Mo—S distance.^[^ ^24^ ^]^ Moreover, the A_1g_ mode exhibits an obvious redshift, which is related to the carrier doping effect, meaning the strong combination of Ti_3_C_2_T*_x_* and MoS_2_ in self‐rolling rod structure.^[^ ^17^, ^25^ ^]^ All these results show that MoS_2_ are formed between the layers of Ti_3_C_2_T*_x_*, which can induce the rolling of Ti_3_C_2_T*_x_* sheets through a strong combination.

To directly observe the interface of Ti_3_C_2_T*_x_* and MoS_2_, a single Ti_3_C_2_T*_x_*/MoS_2_ self‐rolling rod was chosen to prepare a cross‐sectional TEM sample through the focus ion beam (FIB) “lift‐out” technique.^[^ ^26^ ^]^ A TEM sample with a length of about 8 µm was cut by FIB along the axis of the Ti_3_C_2_T*_x_*/MoS_2_ self‐rolling rod (Figure 3c). The detailed preparation process is shown in Figure S10 in the Supporting Information. A typical layered structure can be seen clearly from the cross‐sectional TEM image of the Ti_3_C_2_T*_x_*/MoS_2_ self‐rolling rod, compared with 2D Ti_3_C_2_T*_x_* and 2D Ti_3_C_2_T*_x_*/MoS_2_ sheets (Figure 3d and Figures S6 and S7, Supporting Information). The MoS_2_ layers alternate with Ti_3_C_2_T*_x_* layers with a bending feature, suggesting the rolling of Ti_3_C_2_T*_x_*/MoS_2_ (Figure 3e,f). The thickness of the MoS_2_ layer is about 10 nm and there are typical triangle MoS_2_ crystals with single layer property, which is consistent with the difference of E^1^_2g_ and A_1g_ modes of Ti_3_C_2_T*_x_*/MoS_2_ self‐rolling rod structures in Raman spectra (Figure 3h). An interface zone can be seen clearly between Ti_3_C_2_T*_x_* and MoS_2_, where can be found lattice stripes of both MoS_2_ and Ti_3_C_2_T*_x_*. This means the tight combination of Ti_3_C_2_T*_x_* and MoS_2_. The alternation of MoS_2_ and Ti_3_C_2_T*_x_* would provide abundant nanoheterogeneous interfaces, which can enhance the interfacial polarization loss. The area of heterogeneous interfaces can be calculated according to the number of rolling layers of the Ti_3_C_2_T*_x_*/MoS_2_ self‐rolling rod structure. In an ideal situation, it is an exponential function (Figure S11, Supporting Information). The area can be doubled at maximum, which can realize the full utilization of surface areas of Ti_3_C_2_T*_x_*.

### 2.2 Electromagnetic Response Behavior and EMA Performance

The enhancement of interfacial polarization loss can influence the permittivity of corresponding samples. Generally, the *ε*′ represents the ability to store charge, which is related to the multipolarizations. The *ε*′′ represents the ability to attenuate EM wave energy, which is associated with both polarization loss and conductive loss. As shown in Figure 3b, the *ε*′ of Ti_3_C_2_T*_x_* and Ti_3_C_2_T*_x_*/MoS_2_ sheets are the lowest due to weak interfacial polarization.^[^ ^27^ ^]^ For Ti_3_C_2_T*_x_*/MoS_2_ self‐rolling rods, the *ε*′ increased about two times which is corresponding to the strong interfacial polarization. As to *ε*′′, the trend is the opposite with *ε*′. The *ε*′′ of Ti_3_C_2_T*_x_* and Ti_3_C_2_T*_x_*/MoS_2_ sheets are the highest due to high conductive loss. For Ti_3_C_2_T*_x_*/MoS_2_ self‐rolling rods, the conductive network of Ti_3_C_2_T*_x_* would be interrupted by MoS_2_ inevitably, leading to the decrease of *ε*′′. Also, the electrical conductivity decreased due to the broken conductive network (Figure S16, Supporting Information). It is noted that the *ε*′′ values rise at 9–10 GHz in Ti_3_C_2_T*_x_*/MoS_2_ self‐rolling rods. This is caused by the dielectric relaxation originating from interfacial polarization. The *Cole‐Cole* circles of Ti_3_C_2_T*_x_*/MoS_2_ self‐rolling rod S2 (Figure S14, Supporting Information) reveal that the polarization loss at 9–10 GHz is the strongest. Figure 3i shows the attenuation constant of all samples, which can represent the overall EM loss capacity of the material. It can be found that Ti_3_C_2_T*_x_*/MoS_2_ sheet has a higher EM loss capacity than the Ti_3_C_2_T*_x_*/MoS_2_ self‐rolling rod. However, according to Figures 3j,k, the self‐rolling rod structure shows a larger impedance match area. To better understand this phenomenon, the polarization loss and conductive loss are calculated based on Debye theory (Figure 3l, detailed information is shown in the Supporting Information). The conductive loss plays a primary role in Ti_3_C_2_T*_x_* and Ti_3_C_2_T*_x_*/MoS_2_ sheets due to the high conductivity of Ti_3_C_2_T*_x_*. After the rolling process, the polarization loss increases about two times and plays a primary role with a better impedance match. The better impedance condition of Ti_3_C_2_T*_x_*/MoS_2_ self‐rolling rod structures leads to better EMA performance, compared with Ti_3_C_2_T*_x_*/MoS_2_ sheets in Figure 3m.

**Figure 4:**
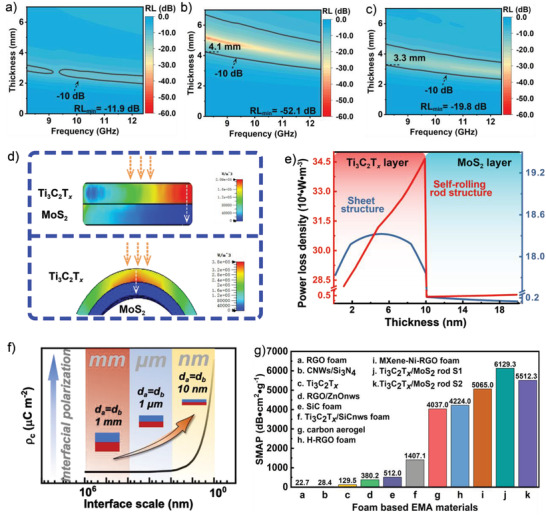
The 2D contours of reflection coefficient versus frequency and thickness (0–7 mm) of a) Ti_3_C_2_T*_x_*/MoS_2_ sheet, b) Ti_3_C_2_T*_x_*/MoS_2_ self‐rolling rod S1, and c) Ti_3_C_2_T*_x_*/MoS_2_ self‐rolling rod S2. d) The distribution of power loss density of sheet structure and rod structure on the heterogeneous interface. e) The value difference of power loss density for sheet structure and rod structure extracted from the white line. f) The relationship of interface scale and interfacial polarization loss (the abscissa axis is logarithm). g) Comparison of the SMAP of the Ti_3_C_2_T*_x_*/MoS_2_ foams in this work with the reported foam‐based EMA materials.

The EMA performances of these foams are evaluated by the wave‐guide method, which ensures the structural integrity of the foam structures. The test direction is vertical to the layer of foams, as shown in Figure S12 in the Supporting Information. This direction maximizes the effect of the layer‐by‐layer structure constructed in the unidirectional freeze‐drying process. Strong reflection of the EM wave caused by the highest *ε*′′ makes Ti_3_C_2_T*_x_* foams show EMI shielding performance (Figure S13, Supporting Information). The downward trend of *ε*′′ with frequency increasing agrees well with the decrease of SE_R_. With the addition of MoS_2_, the EMA performances are improved substantially. The reflection loss (RL) contours of the Ti_3_C_2_T*_x_*/MoS_2_ sheet are shown in **Figure** **4**a. The minimum reflection loss (RL_min_) is −8.2 dB. Although its absorption of EM wave energy reached more than 84%, the narrow absorption band still cannot meet the requirement. In contrast, when Ti_3_C_2_T*_x_*/MoS_2_ sheets are rolled into rods, the RL_min_ can reach −52.1 dB and the EAB can cover the whole *X* band (Figure 4b,c). More detailed RL curves with different thicknesses are shown in Figure S15 in the Supporting Information. The corresponding frequency of RL_min_ shifts to a lower frequency with increased thickness. This effect is caused by the quarter‐wavelength model.^[^ ^28^ ^]^ Due to the excellent absorption of Ti_3_C_2_T*_x_*/MoS_2_ self‐rolling rod‐based foams at the *X* band, the EMA performance at 2–18 GHz was also simulated by the CST microwave studio suite based on the standard finite difference time domain method. The EAB is 6.5 GHz and can cover *C*, *X*, and *Ku* bands (4–18 GHz) with varied thicknesses (Figure S17, Supporting Information). The Ti_3_C_2_T*_x_*/MoS_2_ self‐rolling rod‐based foams can satisfy the application requirements of “thin, light, strong, and broad” for EMA materials. Certainly, the EMA performance could be further improved through tuning the properties of the self‐rolling rod, such as diameters and rolling methods.

### 2.3 EMA Mechanism and Physical Model

To further reveal the advantage of self‐rolling rod structure, the Ti_3_C_2_T*_x_*/MoS_2_ sheet structure and Ti_3_C_2_T*_x_*/MoS_2_ self‐rolling rod structure models are constructed in the CST microwave studio (Figure S20, Supporting Information). The power loss density (PLD) is used to evaluate the EMA absorption ability in different structures. Two lines passing through the highest values of PLD are chosen as representatives. Compared with sheet structure, the self‐rolling rod structure shows higher EMA absorption ability at the heterogeneous interface area (Figure 4e and Figure S18, Supporting Information). Apart from that, the PLD values of the self‐rolling rod structure are much higher than the sheet structure, which can lead to better EMA performance. The Maxwell–Wagner theory also can be used to describe the interfacial polarization loss (Figure S19, Supporting Information).^[^ ^29^ ^]^ The intensity of interfacial polarization loss can be expressed by interfacial charge accumulated density *ρ*(*t*)(*μ*Cm^−2^)^[^ ^30^ ^]^

(1) $$\rho (t)=\frac{\varepsilon_{A}\sigma_{B}-\varepsilon_{B}\sigma_{A}}{\sigma_{A}d_{B}+\sigma_{B}d_{A}}U_{0}(1-e^{-\frac{t}{\tau }})$$

(2) $$\tau =\frac{d_{A}\varepsilon_{B}+d_{B}\varepsilon_{A}}{d_{A}\sigma_{B}+d_{B}\sigma_{A}}$$

where *ε*_A_ and *ε*_B_ are permittivity of A phase with low conductivity and B phase with high conductivity, *d*_A_ and *d*_B_ are the thickness of A and B phases, *U*_0_ is the activation energy and *τ* is the relaxation time. When *d*_A_ = *d*_B_, Equation (2) can be simplified to the following equation

(3) $$\tau =\frac{\varepsilon_{B}+\varepsilon_{A}}{\sigma_{B}+\sigma_{A}}$$

Higher *ρ*(*t*) means stronger interfacial polarization loss. For two certain kinds of materials with similar thickness, the *ρ*(*t*) is only related to interfacial thicknesses *d*_A_ and *d*_B_. As a result, the thinner the interface, the higher the interfacial polarization loss. For the Ti_3_C_2_T*_x_*/MoS_2_ self‐rolling rod structure, the thickness of interfaces was only about 10 nm, which lead to the remarkable improvement of polarization loss. Apart from that, the work functions can affect the accumulation of charges at heterogeneous interfaces, which is related to interfacial polarization loss. The work function difference between Ti_3_C_2_T*_x_* and few‐layer MoS_2_ is higher than bulk MoS_2_ (Figure S21, Supporting Information), which indicates the interfacial polarization loss of Ti_3_C_2_T*_x_*/MoS_2_ self‐rolling rod structure is stronger than sheet structure.^[^ ^31^ ^]^ The work function of few‐layer MoS_2_ is higher than Ti_3_C_2_T*_x_*, which means that the polarized charges will flow to MoS_2_ from Ti_3_C_2_T*_x_*. As a result, the ratio of *ε*_p_′′ is higher than 80%, which surpasses other EMA materials (Table S2, Supporting Information). The high ratio of *ε*_p_′′ endow our samples with superior EMA performance. For a more comprehensive evaluation, the specific EM absorption performance (SMAP), which includes RL, density, and thickness at the same time, is used to evaluate the performance of our samples. The SMAP of Ti_3_C_2_T*_x_*/MoS_2_ foams is higher than 6100 dB cm^2^ g^−1^, due to highly ordered structure and effective interfacial polarization loss after the addition of MoS_2_. Besides, other foam‐based EMA materials with EAB at *X* band are summarized and their corresponding SMAP is shown in Figure 4g and Table S3 in the Supporting Information. The SMAP of Ti_3_C_2_T*_x_*/MoS_2_ self‐rolling rod foams is superior to these reported foam‐based materials,^[^ ^7^, ^32^ ^]^ demonstrating the advantages of self‐rolling rod structures.

## 3 Conclusions

In this work, the different surface tension directions of ATM and Ti_3_C_2_T*_x_* derived from the varied hydrophilic properties induce the self‐rolling of Ti_3_C_2_T*_x_*/MoS_2_ rod structures. According to the simulation and Maxwell–Wagner theory, interfacial polarization loss significantly increases in self‐rolling rod structures. Based on the calculation results of Debye theory, the polarization loss plays a primary role with a ratio of higher than 80% in Ti_3_C_2_T*_x_*/MoS_2_ rod structures. In other words, the interfacial polarization loss is more important than its conduction loss for EMA performance in this system. As a result, the Ti_3_C_2_T*_x_*/MoS_2_ rod structures show better EMA performance compared with Ti_3_C_2_T*_x_*/MoS_2_ sheets, which can realize effective absorption at the whole *X* band. The SMAP is higher than 6100 dB cm^2^ g^−1^ with a density of 0.009 g cm^−3^. A self‐rolling rod structure based on Ti_3_C_2_T*_x_*/MoS_2_ was proposed to realize high interfacial polarization loss for obtaining high‐performance EMA materials. This work provides a new insight to design excellent foam‐based EMA materials with self‐rolling rod structures.

## 4 Experimental Section

### Preparation of Ti_3_C_2_T*_x_* MXenes

Ti_3_C_2_T*_x_* was prepared according to an in situ acid‐etching method. Typically, LiF (1 g, Aladdin materials, China) and HCl (10 mL, 9 m, Aladdin materials, China) were mixed. 2 g of Ti_3_AlC_2_ powder (Jilin 11 technology, China) was added into the mixed solution slowly, following stirring at 40 °C for 24 h. Then, the etching products were collected through centrifugation at 3500 rpm until the supernatant became clear. After that, the mixture was washed about ten times with handshaking, until the supernatant became black. The black Ti_3_C_2_T*_x_* supernatant was collected. The concentration of the suspension was evaluated through the vacuum filtration method.

### Preparation of Ti_3_C_2_T*_x_*/MoS_2_ Foam

Firstly, the concentration of the Ti_3_C_2_T*_x_* suspension was concentrated to 3, 4, and 5 mg mL^−1^, respectively. Then, 0.03 g ATM (Aladdin materials, China) was added to the above solution and sonicated for 30 min for uniform dispersion. After that, the solution was freeze‐dried for 48 h to form the porous structure.^[^ ^33^ ^]^ Finally, the as‐prepared foams were calcined for 2 h at 350 °C under 90% Ar and 10% H_2_. The samples were named S1, S2, and S3, corresponding to the concentration of 3, 4, and 5 mg mL^−1^, respectively. The pure Ti_3_C_2_T*_x_* foam was 5 mg mL^−1^. The Ti_3_C_2_T*_x_*/MoS_2_ foams composed of Ti_3_C_2_T*_x_*/MoS_2_ sheets were also prepared. Typically, the ATM were annealed at first and then mixed with Ti_3_C_2_T*_x_* suspension of 5 mg mL^−1^, followed by the same unidirectional freezing and annealing processes.

### Microstructure Characterization

The structures of foams were characterized by tabletop microscopes (TM400plus, Hitachi, Japan). The morphology of the Ti_3_C_2_T*_x_*/MoS_2_ rod was characterized by SEM (Sigma 300, Zeiss, Germany). The preparation TEM sample through FIB proceeded on FIB (Helios G4 CX, FEI, USA). The distribution of MoS_2_ on the Ti_3_C_2_T*_x_* sheets was observed by Double Cs Corrector TEM (Themis Z, FEI, USA). XRD (D8 Advance, Bruker, Germany) and Raman (He‐Ne laser, *λ* = 532 nm Renishaw, UK) were exploited to analyze the phase compositions. The chemical bonds were characterized by XPS (K‐Alpha, Waltham, Thermo Scientific, USA). TG curves of ATM were obtained on simultaneous thermal analysis (STA 449F3, Netzsch, Germany) in the temperature range of 30–400 °C under Ar.

### Measurement and Calculation of EMA Performance

The S‐parameters and permittivity of the final foams were obtained through the wave‐guide method (ASTM D5568‐14) at a frequency range of 8.2–12.4 GHz. Based on the permittivity, the RL (dB) was obtained through the following equations

(4) $$Z_{\mathrm{in}}=\sqrt{\frac{\mu }{\varepsilon }}\tanh\left[j\left(\frac{2\pi fd}{c}\right)\sqrt{\mu \varepsilon }\right]$$

(5) $$\mathrm{RL}=20\mathrm{lo}g_{10}\left|\frac{Z_{\mathrm{in}}-1}{Z_{\mathrm{in}}+1}\right|$$

where *Z*_in_ is the normalized impedance, and *μ*,*ε*, *c*, *f*, and *d* are permeability, permittivity, speed of light in vacuum, frequency of the EM wave, and the thickness of the test samples. The corresponding frequency range of RL lower than −10 dB is called EAB. The SMAP is adopted as one evaluation index, integrating with density, thickness, RL values, and effective bandwidth. The SMAP values are calculated by the following equation

(6) $$\mathrm{SMAP}(\mathrm{dB}cm^{2}g^{-1})=\frac{\left|\int_{f_{a}}^{f_{b}}\mathrm{RL}df\right|}{f_{b}-f_{a}}/\left(\rho \cdot d\right)$$

where *f*_a_ is the start frequency (8.2 GHz) and*f*_b_ is the stop frequency (12.4 GHz), *d* is the thickness, and *ρ*is the bulk density of the foam.

## Conflict of Interest

The authors declare no conflict of interest.

## Supporting information

Supporting Information

## Data Availability

Research data are not shared.
