# Supplementary material for: Ti3C2T x /MoS2 Self‐Rolling Rod‐Based Foam Boosts Interfacial Polarization for Electromagnetic Wave Absorption
Source: Adv Sci (Weinh). 2022 Apr 11;9(16):2201118. doi: 10.1002/advs.202201118 (PMC9165497; doi:10.1002/advs.202201118)
Supplement: Supplementary file 1 — Supporting Information [file ADVS-9-2201118-s001.pdf]

$Ti_3C_2T_x/MoS_2$ self-rolling rod-based foam boosts interfacial polarization for electromagnetic wave absorption

*Minghang Li, Wenjie Zhu, Xin Li, Hailong Xu, Xiaomeng Fan**, *Hongjing Wu**, *Fang Ye, Jimei Xue, Xiaoqiang Li, Laifei Cheng, Litong Zhang*

Dr. M. Li, Dr. W. Zhu, Dr. X. Li, Prof. X. Fan, Prof. F. Ye, Prof. J. Xue, Prof. X. Li, Prof. L. Cheng and Prof. L. Zhang

Science and Technology on Thermostructural Composite Materials Laboratory

Northwestern Polytechnical University, Xi'an, 710072, China

Dr. H. Xu.

Institute of Textiles and Clothing

Hong Kong Polytechnic University

Prof. H. Wu

MOE Key Laboratory of Material Physics and Chemistry under Extraordinary School of Physical Science and Technology

Northwestern Polytechnical University, Xi'an 710072, P. R. China

Table S1. The correlation between ratio of interfacial polarization loss (ε_p'') and EMA performance

Materials	Ratio of ε_p''	RL (dB)	EAB (GHz)	Ref.
Graphene/Fe_3O_4/polymer	0.76	-63	6	[1]
C/$NiCo_2O_4$	0.80	-52.7	5.2	[2]
CNT/ZnO	0.25	-20.7	4.2	[3]
RGO/Si_3N_4	0.33	-21	4.2	[4]
$Ti_3C_2T_x$/GO	0.01	-49.1	2.9	[5]

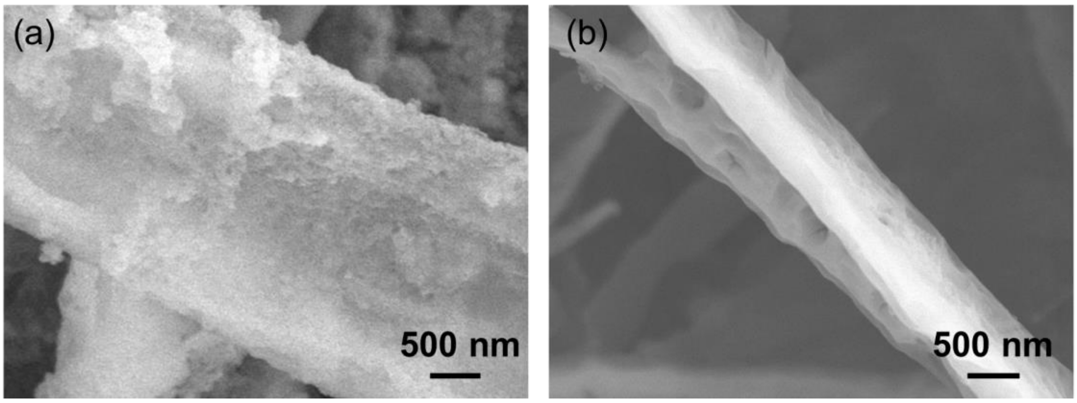

Figure S1. The morphologies of (a) $Ti_3C_2T_x$/ATM and (b) $Ti_3C_2T_x/MoS_2$

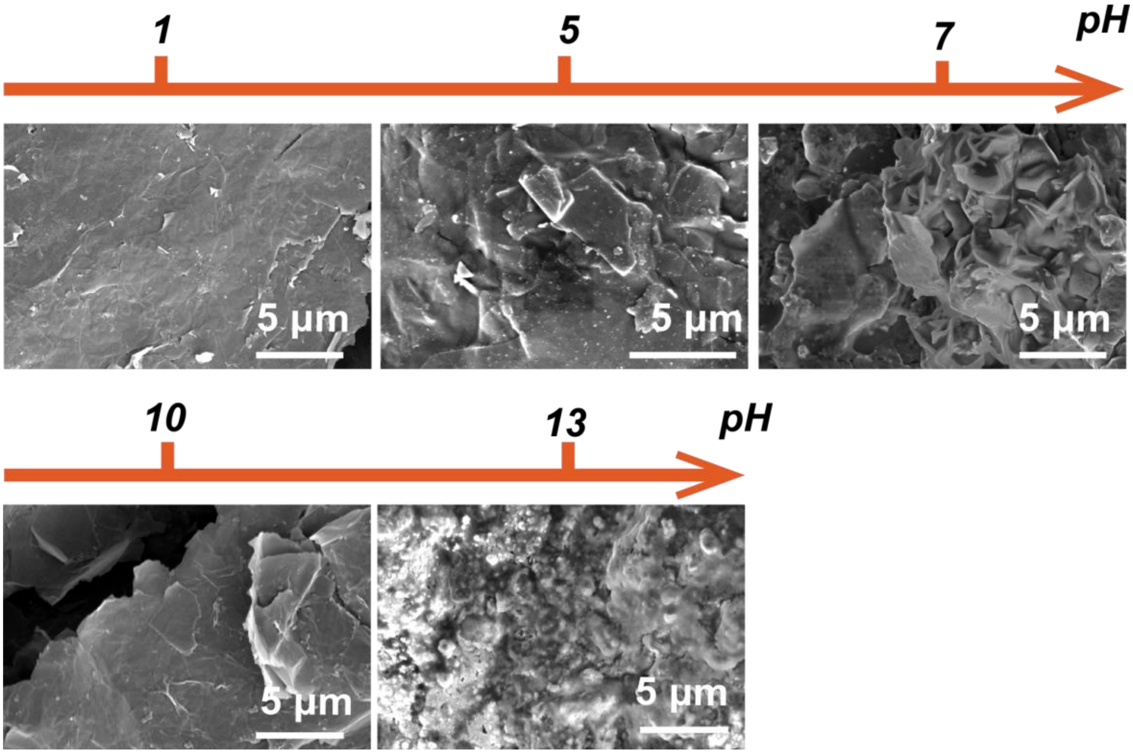

Figure S2. The morphologies of $Ti_3C_2T_x$ sheets with different solutions: (a) HCl, (b) $C_6H_8O_7$, (c) NaCl, (d) NH_4OH and (e) NaOH.

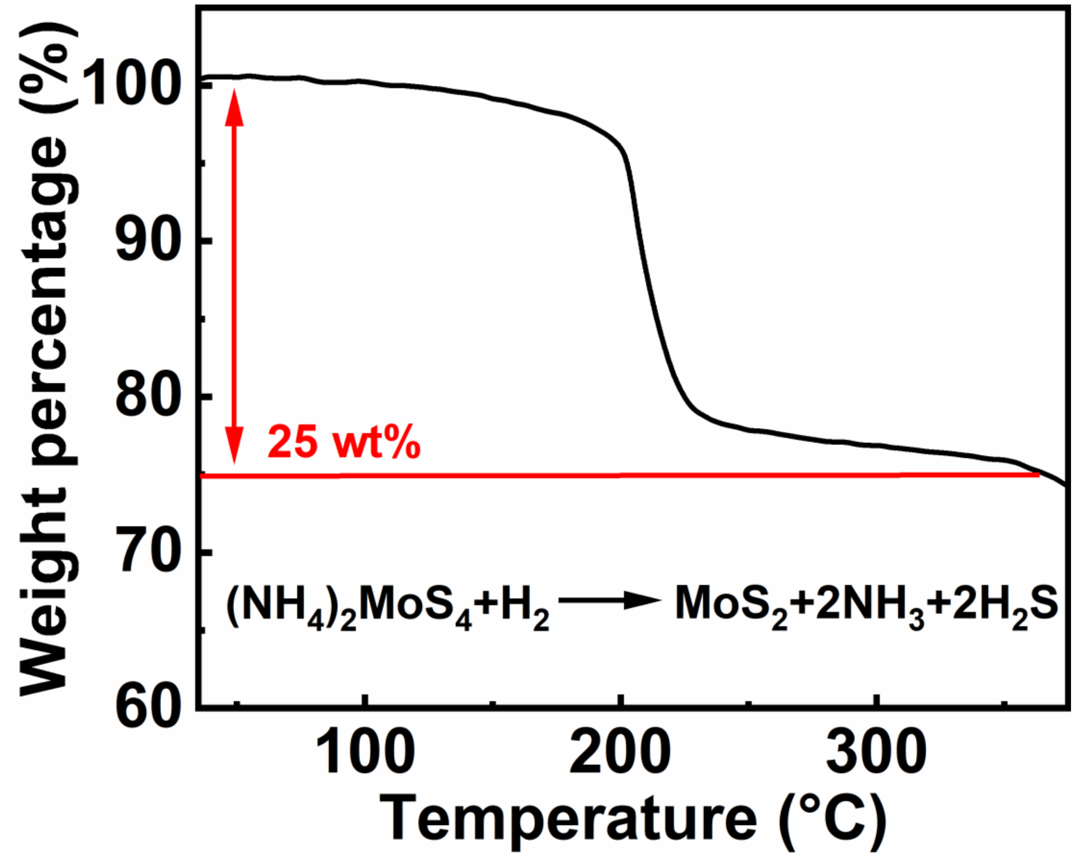

Figure S3. The TG curve of ATM

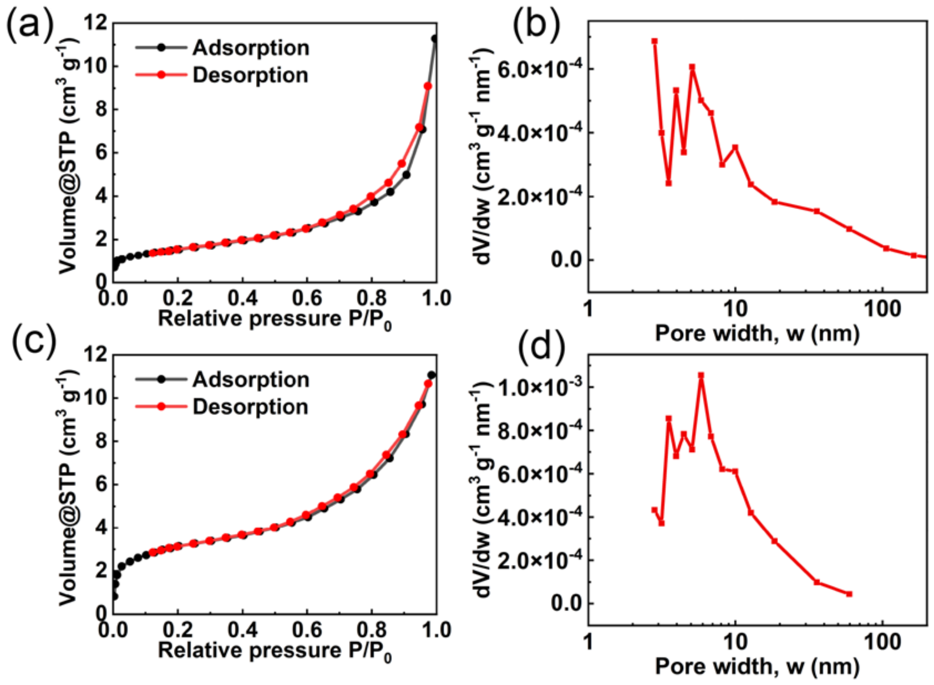

Figure S4. The N_2 sorption isotherms at 77 K and pore size distribution derived from Barrett–Joyner–Halenda (BJH) method of (a, b) $Ti_3C_2T_x/MoS_2$ self-rolling rod structure and (c, d) $Ti_3C_2T_x/MoS_2$ sheet structure.

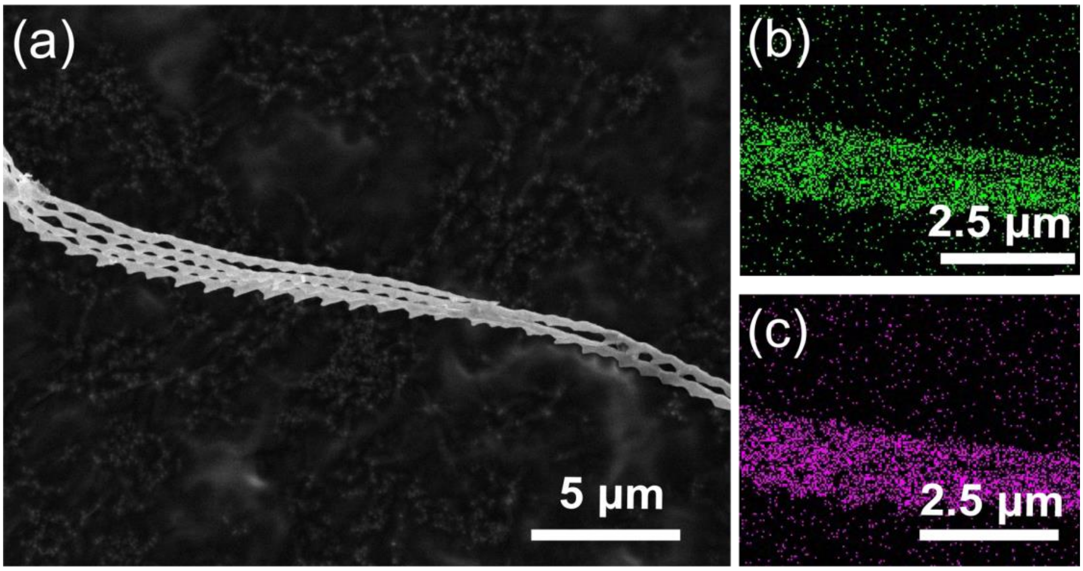

Figure S5. (a) The SEM images of crossed $Ti_3C_2T_x/MoS_2$ rods and (b-c) its corresponding EDS results;

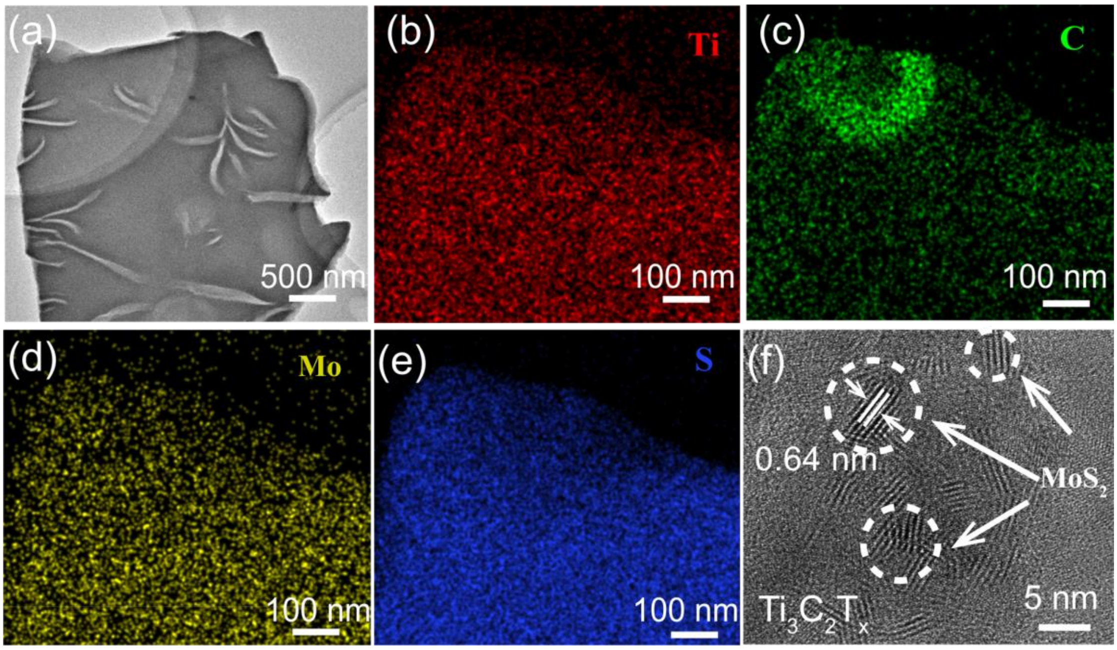

Figure S6. a) The TEM image of sample S2 and b-e) its corresponding EDS results. f) High magnification TEM image of sample S2

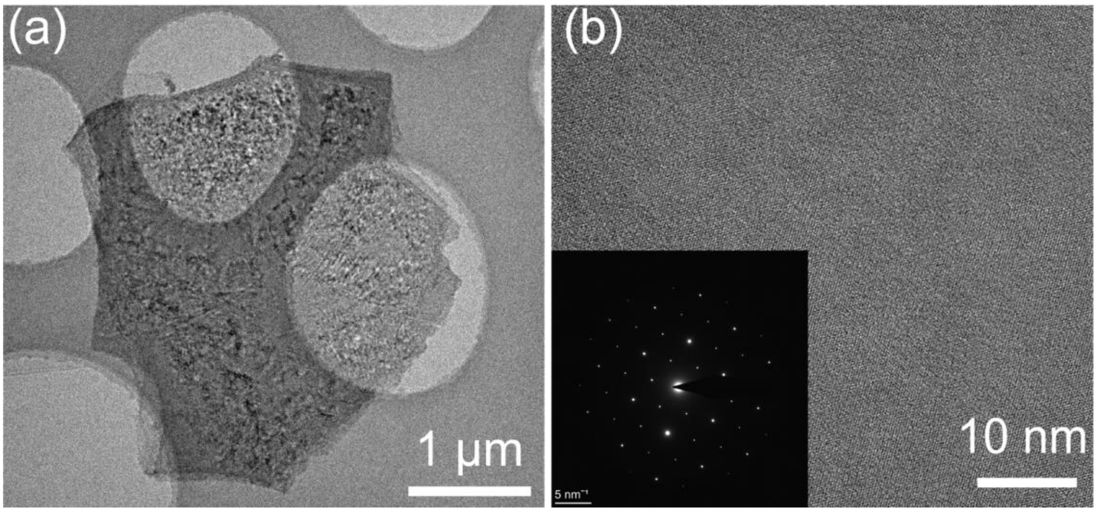

Figure S7. (a) The TEM images of $Ti_3C_2T_x$ and (b) the corresponding high magnification image.

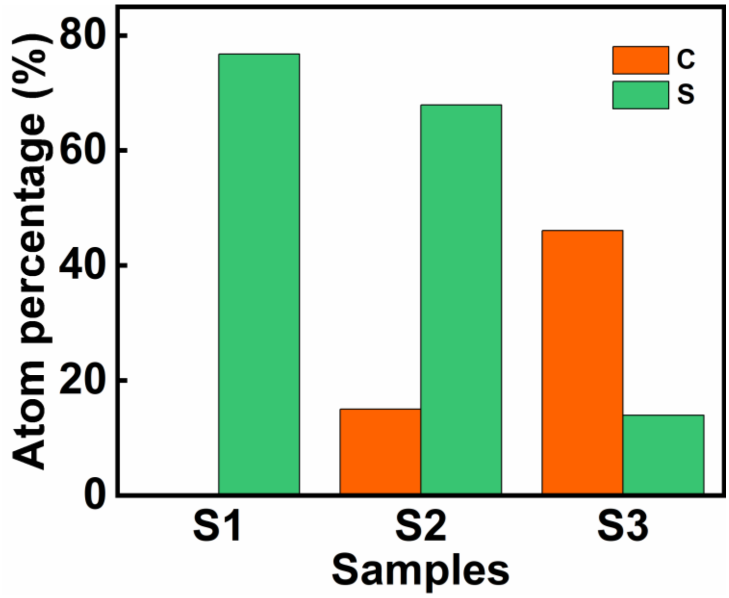

Figure S8. The atom percentage of C and S in different samples.

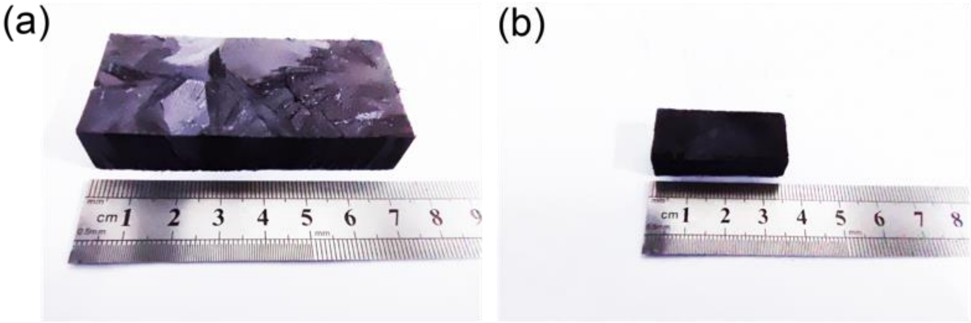

Figure S9. The optical images of foams with sizes of (a) 72 mm *34 mm * 10mm and (b) 35 mm *15 mm *10 mm.

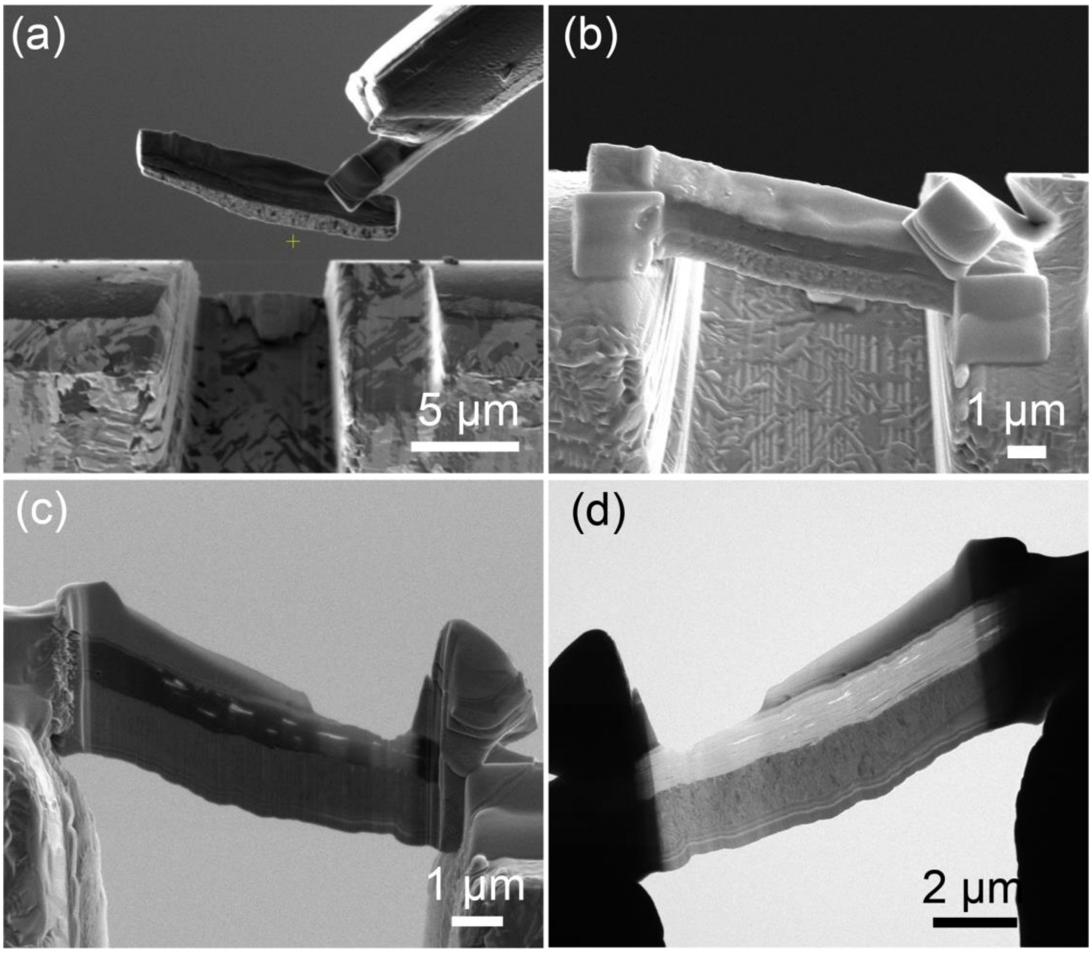

Figure S10. (a) The lift-out process after cutting from $Ti_3C_2T_x/MoS_2$ by the focus ion beam. (b) The free lamella is transferred into the slot position on the TEM half-grid. (c) The SEM image of the final thinned lamella and (d) its corresponding TEM image.

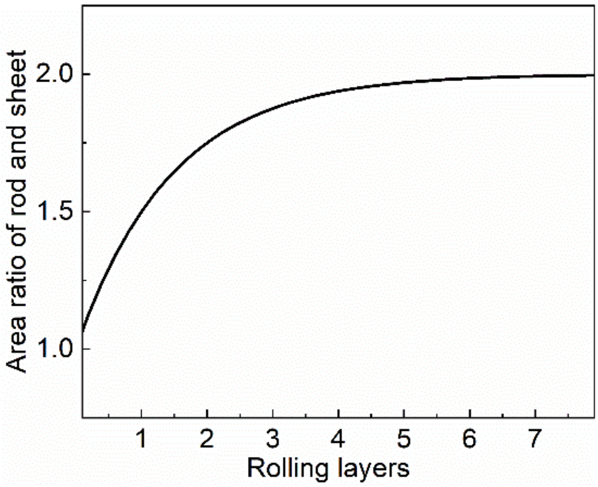

Figure S11. The relationship between rolling layers and area ratio of self-rolling rod structure and sheet structure.

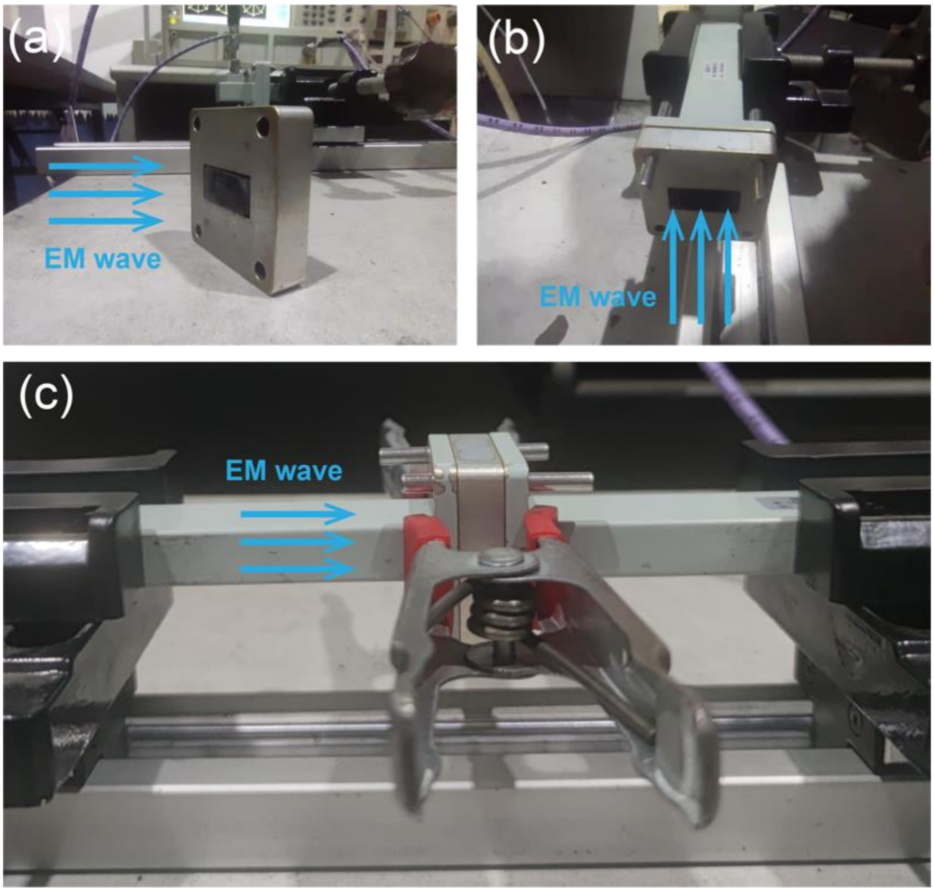

Figure S12. (a) The sample in flange. (b) The flange with sample in wave guide cavity. (c) The test image of wave guide method.

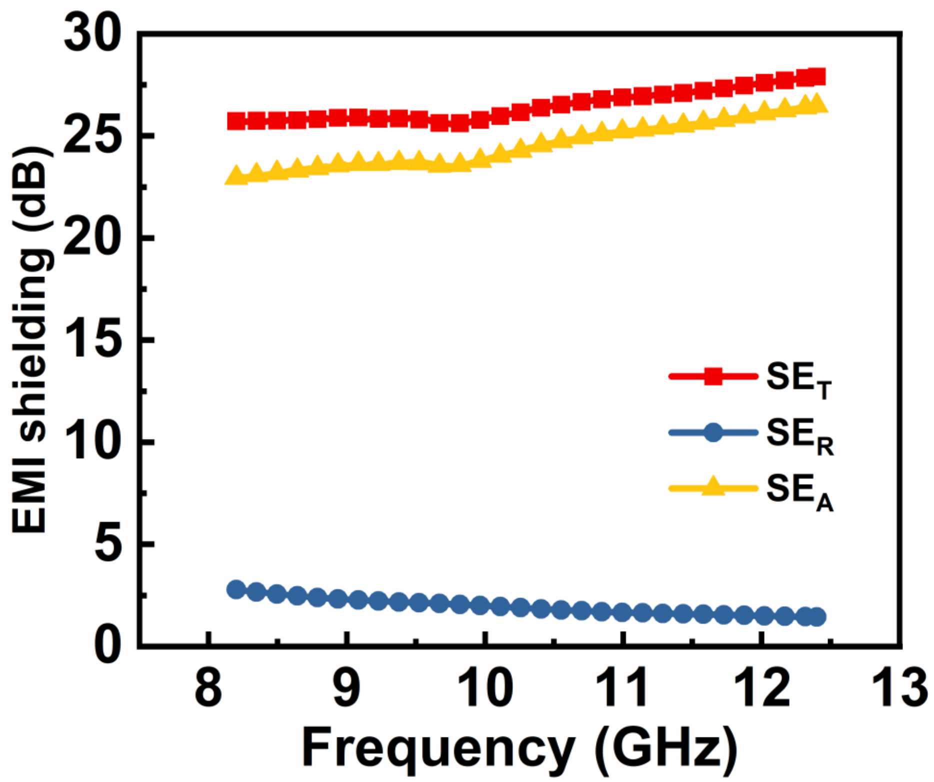

Figure S13. The EMI shielding efficiency of $Ti_3C_2T_x$ foam with a density of 5 mg cm^{-3}

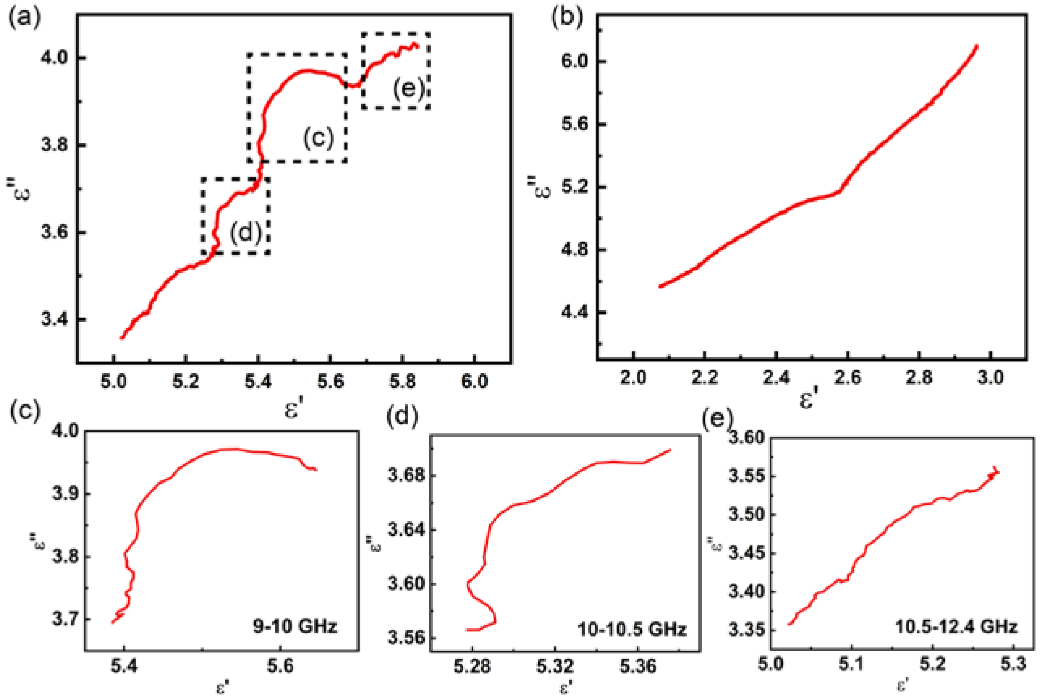

Figure S14. The *Cole-Cole* plots of (a) $Ti_3C_2T_x/MoS_2$ self-rolling rod and (b) $Ti_3C_2T_x/MoS_2$ sheet. (c-e) The magnification plots of inset Figure S14a

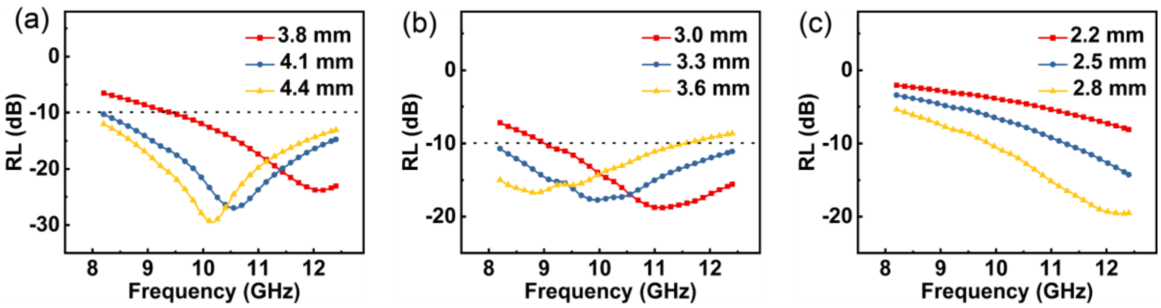

Figure S15. The RL curves with different thickness of samples (a) S1, (b) S2 and (c) S3.

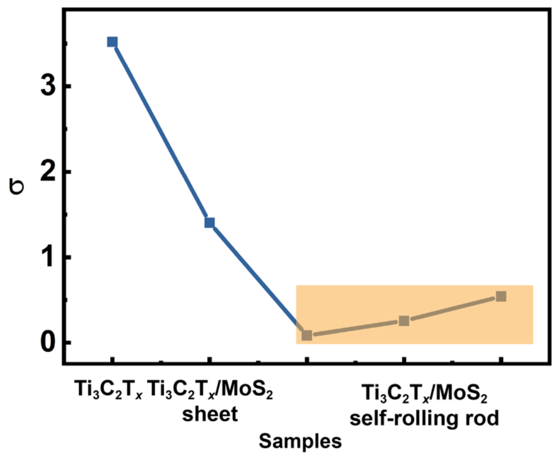

Figure S16. The electrical conductivity of all samples.

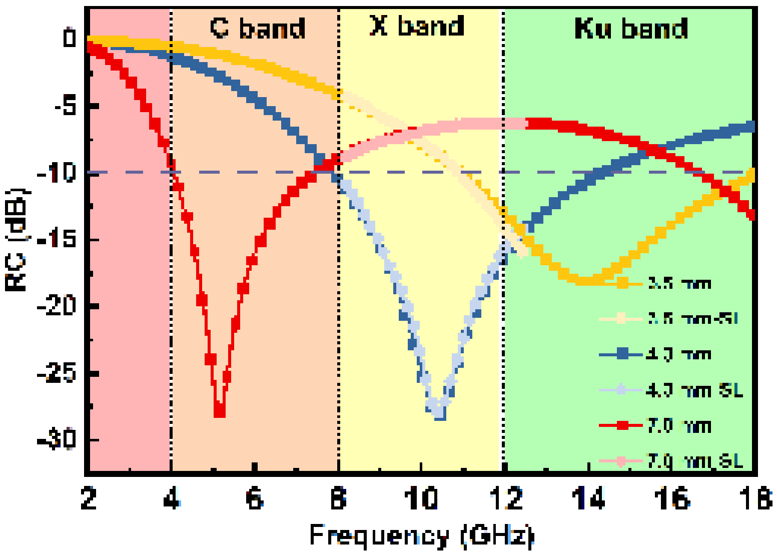

Figure S17. The simulated EMA performance in 2-18 GHz based on the results of CST microwave studio

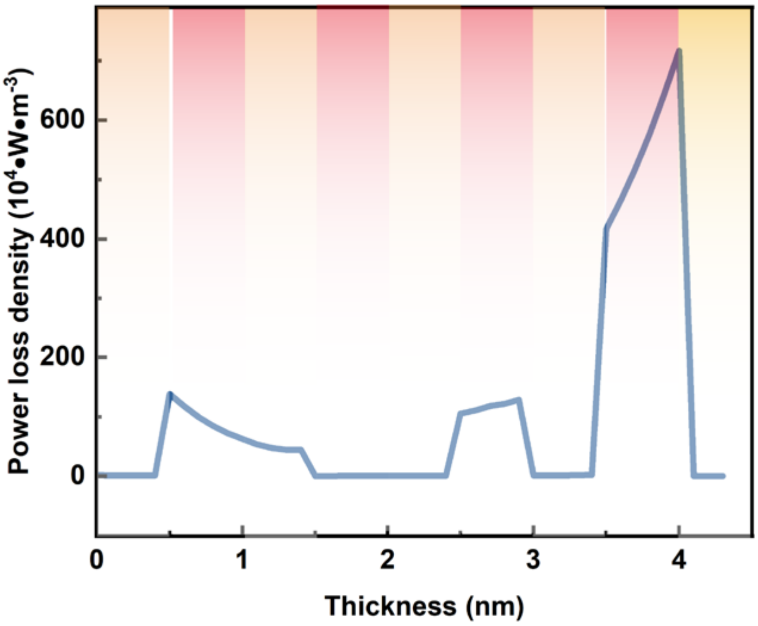

Figure S18. The distribution of power loss density in nine-layer model (red color for MoS_2 and yellow color for $Ti_3C_2T_x$).

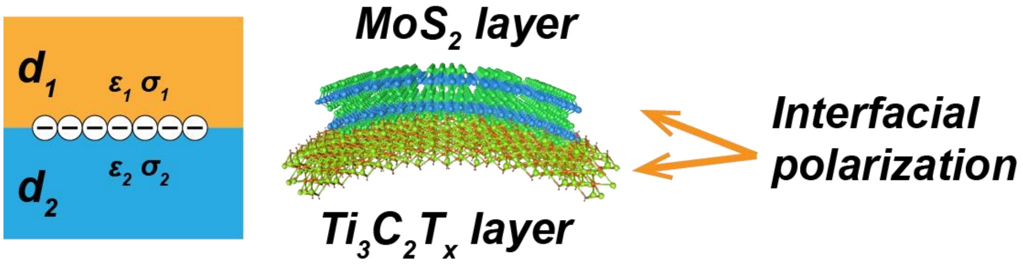

Figure S19. The Maxwell-Wagner effect in $Ti_3C_2T_x/MoS_2$ foams

The simulation of power loss density:

The EM simulation was carried out by using CST microwave studio suite (2020). Periodic structures template with metamaterial-full structure workflow was chose for simulation. Frequency domain solver was used in this simulation process. The overall properties of this simulation are shown in Figure S20a.

To speed up the meshing and simulation process, we scale up the model to millimeter scale (more than 100-million-unit cells must be calculated without scale-up). The wave-guide port was set along the along the layer, which means that the incident direction of EM wave was vertical to the model. The incident direction of EM wave in Figure 4e have been corrected in revised manuscript.

The setup of solver parameters is shown in Figure S20c, tetrahedral mesh type was used to mesh these models.

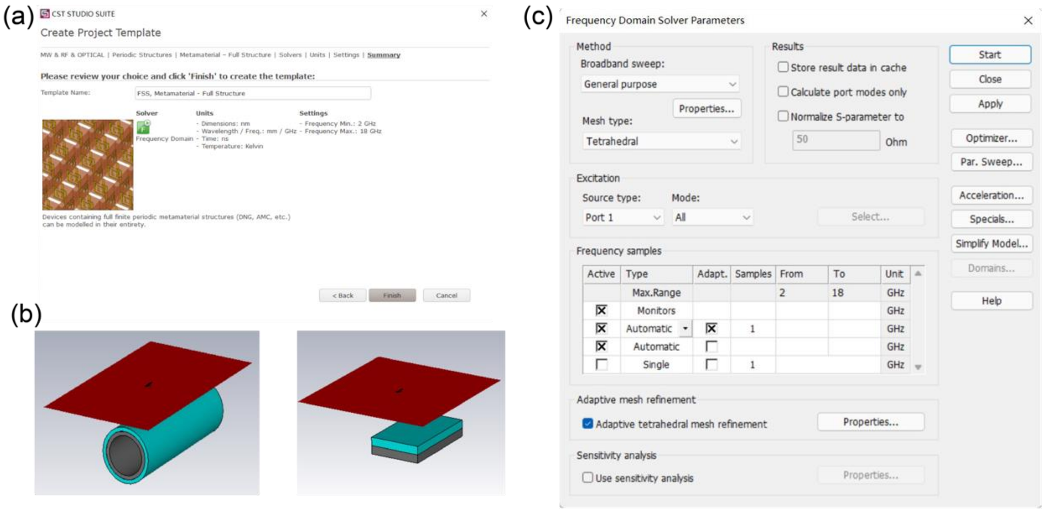

Figure S20. (a) The overall parameters of the simulation project. (b) The model of rod structure and sheet structure in CST microwave studio. (c) The setup of solver parameters.

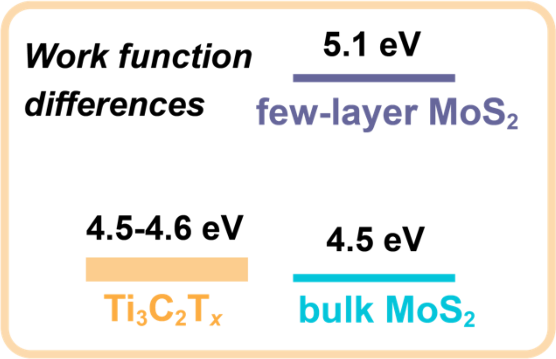

Figure S21. The work function differences of $Ti_3C_2T_x$, bulk MoS_2 and few-layer MoS_2.

Table S2 Comparison of ratio of polarization loss with recently reported works

Samples	Ration of ε_p''	Refs.
Co_3O_4-MWCNT	~0.25	[6]
Graphene/Fe_3O_4/polymer	~0.76	[1]
$Ti_3C_2T_x$@graphene hydrogel	~0.01	[5]
Red-blood cell like carbon sphere	~0.6	[7]
RGO/Si_3N_4	~0.17	[4]
**$Ti_3C_2T_x$/MoS_2 rod**	**0.88**	**This work**

Table S3 EM absorption performance of representative foam-based absorbers

Samples	Matrix	Density ($mg\cdot cm^{-3}$)	RL≤-10dB d (mm)	RL≤-10dB EAB (GHz)	SMAP ($dB\cdot cm^2\cdot g^{-1}$)	Refs.
$Ti_3C_2T_x/SiC_{nws}$ foam	Air	0.029	3.9	4.2	1407	[8]
$Ti_3C_2T_x$ foam	Paraffin	~0.8	1.4	4.2	129.46	[9]
RGO/ZnO_{nws}	PDMS	>0.097	4.8	4.2	~380.2	[10]
$CNWs/Si_3N_4$	Si_3N_4	~1.97	3.2	4.2	28.4	[11]
RGO/SiC_{nws}	PDMS	>0.097	3	4.2	512	[12]
RGO foam	Paraffin	~0.9	10	4.2	22.7	[13]
MXene-Ni-GO	Air	6.45	2.15	2.4	5065	[14]
H-RGO	Air	6.1	3.4	3.2	4224	[15]
SiC foam	Air	11.56	3.5	1.2	511.97	[16]
Carbon aerogel	Air	6.1	3.5	4.2	4037	[17]
**$Ti_3C_2T_x/MoS_2$ S1**	**Air**	**0.013**	**3.9**	**4.2**	**6129.3**	**This work**
**$Ti_3C_2T_x/MoS_2$ S2**	**Air**	**0.014**	**3.1**	**4.2**	**5512.3**	**This work**

Calculation process of polarization loss and conductive loss:

The calculation process of ε_p'' and ε_c'' is based on Debye theory:

$$\varepsilon' = \varepsilon_\infty + \frac{\varepsilon_s - \varepsilon_\infty}{1+\omega^2\tau^2} \quad (0.1)$$

$$\varepsilon'' = \varepsilon_p'' + \varepsilon_c'' = \frac{\varepsilon_s - \varepsilon_\infty}{1+\omega^2\tau^2}\omega\tau + \frac{\sigma}{\omega\varepsilon_0} \quad (0.2)$$

Non-linear least squares method is used to calculate the ε_p'' and ε_c'' [7]. A Python package Scipy to simplify our code[18]. The detailed code is shown following.

```
import numpy as np
from scipy.optimize import least_squares
import xlrd
def realimag(array):
        return np.array([(x.real, -x.imag) for x in array])
def func(w,p):
    s,u,sigma,t=p
    d=complex(0,1)
    o=8.854187817*10**(-12)
    return realimag(u+(s-u)/(1+np.dot(d,np.dot(w,t)))-np.dot(d,np.divide(sigma,np.dot(w,o))))
def funcresult_conducloss(w,p):
    s,u,sigma,t=p
    o=8.854187817*10**(-12)
    return np.divide(sigma,np.dot(w,o))
def residuals(p, y,x):
    return (realimag(np.array(y)) - func(x, p)).flatten()
p0 = [100,100,30,10**-11]
data=xlrd.open_workbook('permittivity.xlsx')
table=data.sheets()[0]
fsig=0
ft=0
fplsq=[]
fs=0
fu=0
fconducloss=0
fpolarloss=0
fcost=0
for i in range(1,21):
    end=i*10
    start=end-10
```

```
    xdata=table.col_values(0)[start:end]
    ydata_1=table.col_values(2)[start:end]
    ydata_2=table.col_values(1)[start:end]
    ydata=[]
    for i in range(10):
        ydata.append(complex(ydata_2[i],-ydata_1[i]))
    plsq = least_squares(residuals, p0, bounds=([0,0,0,0],[200,200,100,10**-10]),args=(ydata, xdata),max_nfev=10000000)
    fplsq.append(plsq)
    conducloss=np.mean(funcresult_conducloss(xdata,plsq.x))
    fconducloss+=conducloss
    polarloss=np.mean(ydata_1)-conducloss
    fpolarloss+=polarloss
    fsig=fsig+plsq.x[2]
    fs=fs+plsq.x[0]
    fu=fu+plsq.x[1]
    ft=ft+plsq.x[3]
    fcost=plsq.cost+fcost
print(fs/20)
print(fu/20)
print(fsig/20)
print(ft/20)
print(fpolarloss/20) # polarization loss
print(fconducloss/20) #conductive loss
print(fpolarloss/(fpolarloss+fconducloss))
print(fcost/20)
```

References:

[1] J. C. Shu, M. S. Cao, M. Zhang, X. X. Wang, W. Q. Cao, X. Y. Fang, M. Q. Cao, *Adv. Funct. Mater.* **2020**, 30, 1908299.

[2] C. Han, M. Zhang, W. Cao, M. Cao, *Carbon* **2021**, 171, 953.

[3] M. Lu, W. Cao, H. Shi, X. Fang, J. Yang, Z. Hou, H. Jin, W. Wang, J. Yuan, M. Cao, *J. Mater. Chem.A* **2014**, 2, 10540.

[4] Z. Hou, X. Yin, H. Xu, H. Wei, M. Li, L. Cheng, L. Zhang, *ACS Appl. Mater. Interfaces* **2019**, 11, 5364.

[5] Y. Li, F. Meng, Y. Mei, H. Wang, Y. Guo, Y. Wang, F. Peng, F. Huang, Z. Zhou, *Chem. Eng. J.* **2020**, 391, 123512.

[6] J. Shu, X. Huang, M. Cao, *Carbon* **2021**, 174, 638.

[7] H. Xu, X. Yin, M. Li, F. Ye, M. Han, Z. Hou, X. Li, L. Zhang, L. Cheng, *Carbon* **2018**, 132, 343.

[8] X. Li, X. Yin, H. Xu, M. Han, M. Li, S. Liang, L. Cheng, L. Zhang, *ACS Appl. Mater. Interfaces* **2018**, 10, 34524.
[9] K. Hu, H. Wang, X. Zhang, H. Huang, T. Qiu, Y. Wang, C. Zhang, L. Pan, J. Yang, *Chem. Eng. J.* **2020**, 408, 127283.
[10] C. Song, X. Yin, M. Han, X. Li, Z. Hou, L. Zhang, L. Cheng, *Carbon* **2017**, 116, 50.
[11] H. Pan, X. Yin, J. Xue, L. Cheng, L. Zhang, *Carbon* **2016**, 107, 36.
[12] M. Han, X. Yin, Z. Hou, C. Song, X. Li, L. Zhang, L. Cheng, *ACS Appl Mater Interfaces* **2017**, 9, 11803.
[13] Y. Zhang, Y. Huang, H. Chen, Z. Huang, Y. Yang, P. Xiao, Y. Zhou, Y. Chen, *Carbon* **2016**, 105, 438.
[14] L. Liang, Q. Li, X. Yan, Y. Feng, Y. Wang, H. B. Zhang, X. Zhou, C. Liu, C. Shen, X. Xie, *ACS Nano* **2021**, 15, 6622.
[15] M. Zhang, X. Fang, Y. Zhang, J. Guo, C. Gong, D. Estevez, F. Qin, J. Zhang, *Nanotechnology* **2020**, 31, 275707.
[16] X. Ye, Z. Chen, M. Li, T. Wang, C. Wu, J. Zhang, Q. Zhou, H. Liu, S. Cui, *J. Alloys Compd.* **2020**, 817.
[17] L. Liang, Z. Zhang, F. Song, W. Zhang, H. Li, J. Gu, Q. Liu, D. Zhang, *Carbon* **2020**, 162, 283.
[18] S. v. d. Walt, S. C. Colbert, G. Varoquaux, *Comput. Sci. Eng.* **2011**, 13, 22.
